# High expression of microRNA-130b correlates with poor prognosis of patients with hepatocellular carcinoma

**DOI:** 10.1186/s13000-014-0160-5

**Published:** 2014-08-15

**Authors:** Wen-yao Wang, Hong-fei Zhang, Lei Wang, Yan-peng Ma, Fei Gao, Shao-jun Zhang, Li-chao Wang

**Affiliations:** Department of General Surgery, The Second Hospital of hebei Medical university, Shijiazhuang, Hebei 050000 China

## Abstract

**Background:**

Whether microRNA-130b(miR-130b) can serve as a prognostic biomarker of hepatocellular carcinoma (HCC) has not been investigated. In the present study, we investigated the feasibility of miR-130b as a novel prognostic biomarker for HCC.

**Methods:**

We retrospectively investigated 97 patients diagnosed with HCC who underwent routine curative surgery between May 2007 and July 2012. miR-130b expression in HCC tissues and paired normal adjacent liver tissues was measured by reverse transcription and real-time PCR (RT-PCR). Survival curves were plotted using the Kaplan-Meier method and differences in survival rates were analyzed using the log-rank test.

**Results:**

miR-130b expression level was significantly higher in HCC tissues compared with normal adjacent liver tissues (*P* < 0.0001). The 5-year overall survival (OS) of high miR-130b expression group was significantly shorter than that of low miR-130b expression group (43.6% vs. 71.5%; *P* = 0.022). Moreover, the 5-year disease-free survival (DFS) of high miR-130b expression group was also significantly shorter than that of low miR-130b expression group (25.9% vs. 63.9%; *P* = 0.012). In a multivariate Cox model, we found that miR-130b expression was an independent prognostic factor for both 5-year OS (hazards ratio [HR] = 2.523, 95% confidence interval [CI] = 1.024-7.901, *P* = 0.011) and 5-year DFS (HR = 4.003, CI = 1.578-7.899, *P* = 0.005) in HCC.

**Conclusion:**

The results indicated that high expression of microRNA-130b was correlated with significant characteristics of patients with HCC, and it might be useful as a novel prognostic biomarker for HCC.

**Virtual Slides:**

The virtual slide(s) for this article can be found here: http://www.diagnosticpathology.diagnomx.eu/vs/13000_2014_160

## Background

Hepatocellular carcinoma (HCC) is the third most common cause of mortality from cancer worldwide [1]. In China, HCC is the second highest cancer killer since the 1990s, which alone accounts for 53% of all liver cancer deaths worldwide [2]. More than 90% of HCC cases develop in chronically inflamed liver as a result of viral hepatitis, alcohol abuse and in increasing incidence in patients with non-alcoholic fatty liver disease [3]. Although the clinical staging systems for HCC have been used in routine clinical decision making, there is still a need to refine and complement outcome predictions. Modifications of these staging systems by the addition of new biomarkers are likely to improve the prognostic assessment of HCC patients and could therefore fulfill a clinical need [4-9].

MicroRNAs (miRNAs) are endogenously expressed, small interfering RNAs [10]. They are transcribed as precursor molecules that are subsequently processed into the active ~21 nucleotide mature miRNAs. The mature miRNA binds to the 3’untranslated region of the target mRNA through imperfect base pairing, producing translational arrest and/or degradation of the mRNA. A growing number of both direct and indirect evidence suggests a relationship between differential miRNA expression and cancer [11,12]. miR-130b has been found to be deregulated in some types of cancers, including being overexpressed in gastric cancer [13,14], glioma [15], and renal cell carcinoma(RCC) [16], while being downregulated in endometrial cancer [17] and papillary thyroid carcinoma [18]. Previously, Liu et al. reported that the expression level of miR-130b was upregulated in HCC tissues. Furthermore, circulating miR-130b in serum was a biomarker with clinical value for HCC screening [19]. However, whether miR-130b can serve as a prognostic biomarker of HCC has not been investigated. Therefore, in the present study, we investigated the feasibility of miR-130b as a novel prognostic biomarker for HCC.

## Methods

### Patients and tissue samples

We retrospectively investigated 97 patients diagnosed with HCC who underwent routine curative surgery between May 2007 and July 2012 at The Second Hospital of hebei Medical university. None of the patients received radiotherapy or chemotherapy before surgery. For each case, the diagnosis and the histologic grade were confirmed by two pathologists. The fresh human HCC tissues and paired normal adjacent liver tissues were obtained from each HCC patients. Tissues were snap frozen in liquid nitrogen after surgical resection until use. Followup included serum a-fetoprotein (AFP) level, abdominal ultrasonography, and chest radiography every 1–3 months after curative hepatectomy. When tumor recurrence was suspected, computed tomography scan (CT) or/and magnetic resonance imaging scan (MRI) was performed to confirm the diagnosis. The clinico-pathological features of the patients were summarized in Table 1. The present study was approved by the Research Ethics Committee of The Second Hospital of hebei Medical university. Informed consent was obtained from all the patients. All specimens were handled and made anonymous according to the ethical and legal standards.

**Table 1:** **Clinicopathological features and miR-130b expression in HCC**

		**miR-130b expression level**		
**Clinicopathologic variables**	**Number of cases**	**Low expression**	**High expression**	**P value**
Age				
<50	46	25	21	
≥50	51	23	28	0.81
Gender				
Male	59	29	30	
Female	38	19	19	0.85
HBsAg				
Postive	61	20	41	
Negtive	36	28	8	0.02
AFP (ng/ml)				
<25	48	38	10	
≥25	49	10	39	0.04
Tumor size (cm)				
<5	52	40	12	
≥5	45	8	37	0.04
Histologic grade				
High	33	4	29	
Low	64	44	20	0.005
Tumor number				
Solitary	61	34	27	
Multiple	36	14	22	0.08
Vein invasion				
Presence	36	17	19	
Absence	61	31	30	0.41
TNM stage				
I-II	52	44	8	
III–IV	45	4	41	<0.001

AFP = a-fetoprotein.

### Isolation of total RNA and real-time PCR analysis

MiR-130b expression level in HCC tissues and paired normal adjacent tissues was measured by reverse transcription and real-time PCR (RT-PCR). Total RNA was isolated from frozen samples using Trizol reagent (Invitrogen, CA, U.S.A.) according to the manufacturer’s protocol. The TaqMan microRNA assay and TaqMan universal PCR master mix were used to detect the expression of miR-130b, and the U6 gene was used as an internal control to normalize variances. Relative quantification of target miRNA expression was evaluated using the comparative cycle threshold (CT) method. Each sample was examined in triplicate and the raw data were presented as the relative quantity of target miRNA, normalized with respect to U6.

### Statistical analysis

The Mann–Whitney test or Kruskal–Wallis was performed to determine the significance of miRNA levels. Survival curves were plotted using the Kaplan-Meier method and differences in survival rates were analyzed using the log-rank test. Prognostic relevance of each variable to overall survival (OS) and disease-free survival (DFS) were analyzed using the Cox regression model. Multivariate analysis of the prognostic factors was performed with Cox regression model. P < 0.05 was considered statistically significant. All statistical calculations were performed using SPSS 18.0 for Windows (SPSS Inc, IL, USA).

## Results

### miR-130b was significantly upregulated in HCC tissues

**Figure 1:**
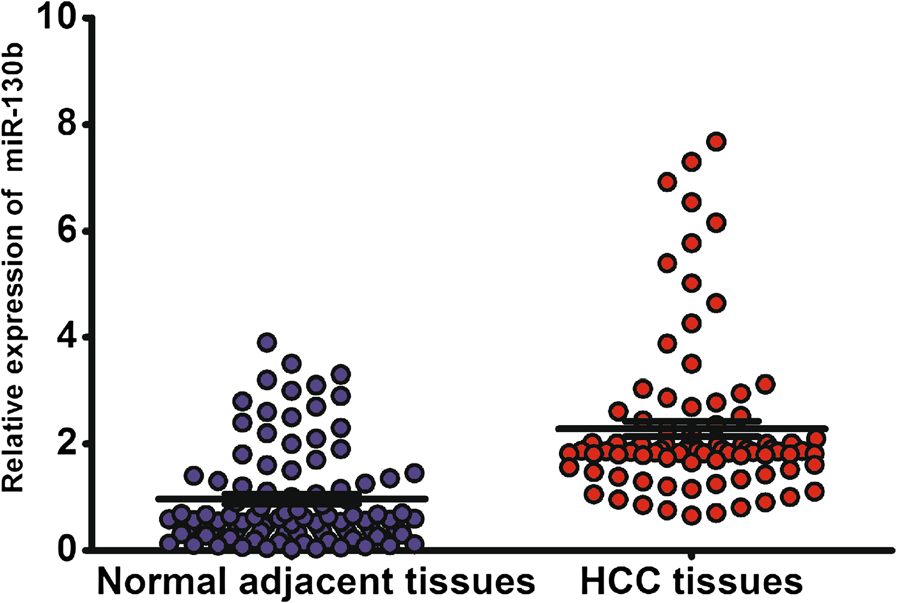
**The relative expression level of miR-130b in human HCC tissues (n = 97) and matched adjacent noncancerous liver tissues (n = 97).** miR-130b expression was significantly higher in HCC tissues (median expression level: 1.90, range 0.65–7.68) compared with normal adjacent liver tissues (median relative expression level: 0.58, range 0.03–3.90; p < 0.0001)

We analyzed the expression levels of miR-130b in 97 pairs of HCC tissues and normal adjacent tissues from 97 HCC patients. As revealed by quantitative RT-PCR analysis, miR-130b expression level was significantly higher in HCC tissues (median expression level: 1.90, range 0.65–7.68) compared with normal adjacent liver tissues (median relative expression level: 0.58, range 0.03–3.90; *P* < 0.0001, Figure 1).

### Association between miR-130b expression and the clinicopathological features of HCC

For better understanding of the clinical relevance of miR-130b expression in HCC, we divided the 97 HCC patients into a high expression group (n =49) and a low expression group (n =48), according to the median expression level of miR-130b (1.90) in all HCC samples. And, the relationships of the miR-130b with various clinical features of HCC were analyzed and summarized in Table 1. The results revealed that a high level of miR-130b expression was correlated with serum a-fetoprotein (AFP) level (P = 0.04), HBsAg status (*P* =0.02), tumor size (*P* = 0.04), high histologic grade (*P* =0.005), and high TNM stage (p < 0.001). However, there were no significant correlations of miR-130b expression with other clinical features such as gender, age, vein invasion, and tumor number(all *P* > 0.05).

### Prognostic values of miR-130b expression in HCC

**Figure 2:**
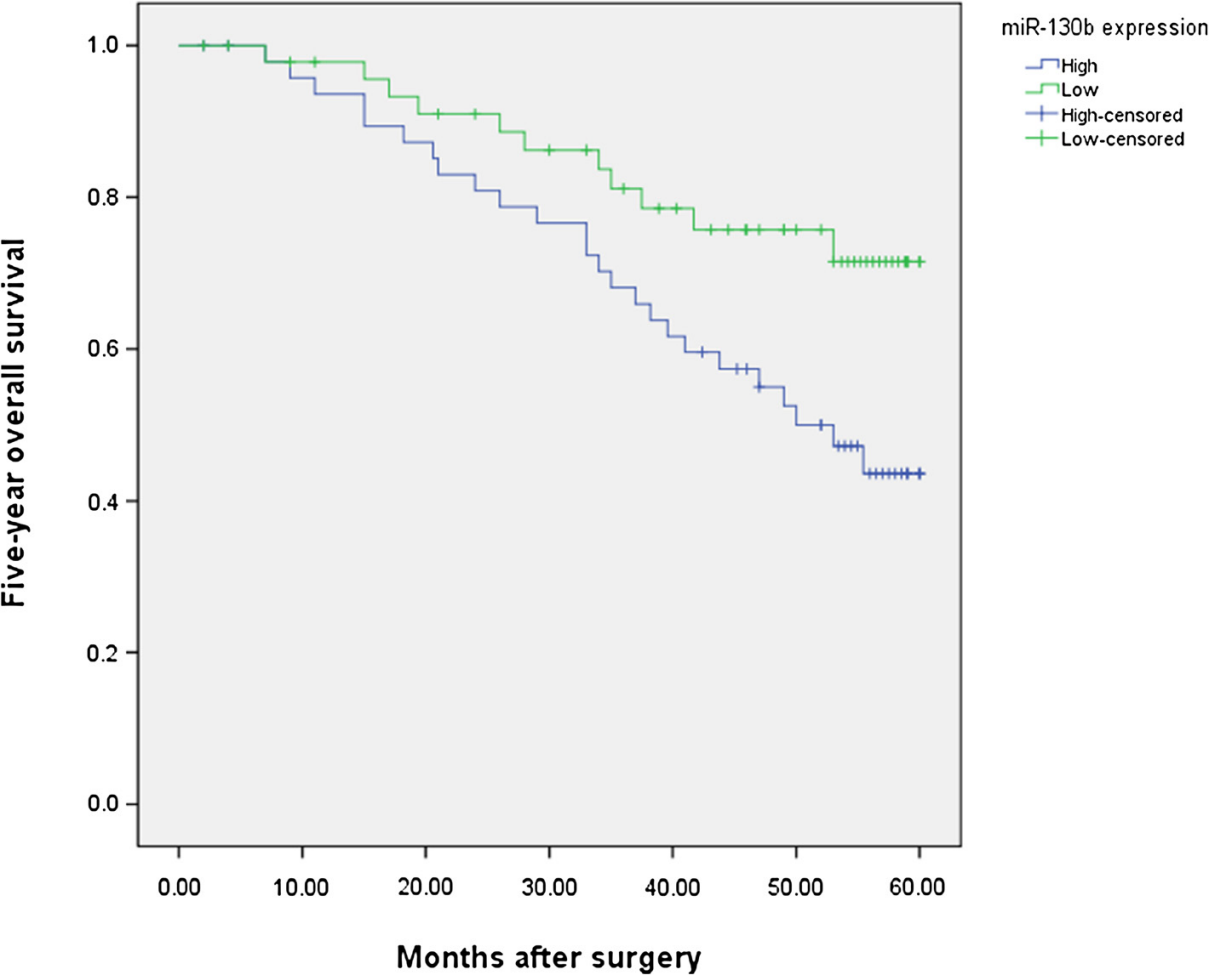
**Kaplan-Meier curves of the overall survival of 97 HCC patients.** Overall survival rate in patients with high miR-130b expression was significantly lower than that in patients with low miR-130b expression.

**Figure 3:**
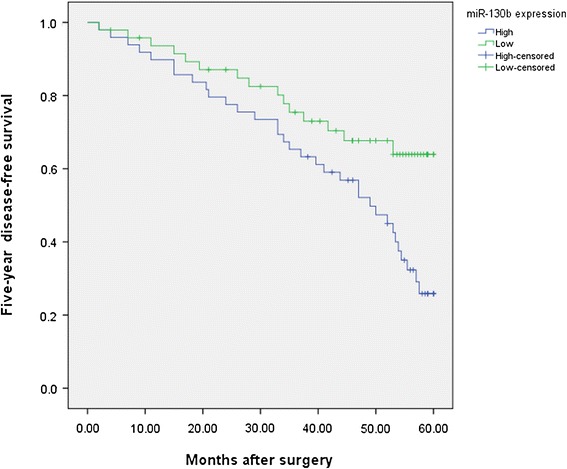
**Kaplan-Meier curves of the disease-free survival of 97 HCC patients.** Disease-free survival rate in patients with high miR-130b expression was significantly lower than that in patients with low miR-130b expression.

To further investigate the correlations of miR-130b expression level with survival of patients with HCC, Kaplan-Meier analyses were performed. As shown in Figure 2, the 5-year OS of high miR-130b expression group was significantly shorter than that of low miR-130b expression group (43.6% vs. 71.5%; *P* = 0.022). Moreover, the 5-year DFS of high miR-130b expression group was also significantly shorter than that of low miR-130b expression group (25.9% vs. 63.9%; *P* = 0.012, shown in Figure 3). Furthermore, in a multivariate Cox model, we found that miR-130b expression was an independent poor prognostic factor for both 5-year OS (hazards ratio [HR] = 2.523, 95% confidence interval [CI] = 1.024-7.901, *P* = 0.011, Table 2) and 5-year DFS (HR = 4.003, CI = 1.578-7.899, *P* = 0.005, Table 2) in HCC.

**Table 2:** **Multivariate analysis of the associations of prognosis with various clinicopathologic parameters and miR-130b expression in HCC patients**

	**Overall survival**			**Disease-free survival**		
Features	HR	95% CI	P value	HR	95% CI	P value
Age	0.981	0.378-1.467	0.41	0.827	0.461-1.251	0.39
Gender	1.091	0.672-1.871	0.56	0.914	0.671-1.889	0.52
HBsAg	1.671	0.871-4.778	0.12	2.121	0.871-5.113	0.14
AFP level	2.119	0.981-5.889	0.11	3.671	0.367-12.112	0.23
Tumor size	3.121	0.478-7.889	0.34	4.116	0.781-11.234	0.31
Histologic grade	4.891	2.113-5.991	0.04	5.995	3.467-8.114	0.01
Tumor number	2.997	0.912-3.091	0.13	3.111	0.872-9.112	0.09
Vein invasion	1.982	0.891-1.971	0.09	2.891	0.781-5.997	0.08
TNM stage	2.131	1.091-6.901	0.009	4.121	1.341-7.113	0.007
miR-130b expression	2.523	1.024-7.901	0.011	4.003	1.578-7.889	0.005

AFP = a-fetoprotein; CI = confidence interval; HR = hazards ratio.

## Discussion

A growing number of novel treatment strategies have been developed for HCC, including molecular targeted therapy, gene therapy, and immunotherapy. However, satisfactory therapeutic outcomes have not been achieved, and the survival rate of HCC is still low. A complete understanding of the molecular mechanisms underlying tumor initiation and progression is essential for novel prognostic and therapeutic approaches aimed at improving the outcome of patients with HCC. Over the last years, miRNAs are emerging as a new class of gene regulators involved in different malignancies.

The miR-130 family is formed by mature miR-130a and miR-130b that share the same seed sequence and are coded by two independent loci (miRBase Database). miR-130 has been found to linked to mesenchymal differentiation, immune cell function, and hypoxic response modulation [20]. miR-130 has also been validated as a peroxisome proliferator–activated receptor γ (PPARγ) regulator because it suppresses the adipogenic process through the binding to two distinct highly conserved sites located in the coding sequence and 3’UTR of the corresponding mRNA [20,21]. miR-130b has been found to be deregulated in some types of cancers, including being overexpressed in gastric cancer [13,14], glioma [15], and RCC [16], while being downregulated in endometrial cancer [17] and papillary thyroid carcinoma [18]. For example, Zhao et al. demonstrated that the deregulated expression of miR-130b was associated with poor prognosis and aggressive phenotype of pancreatic cancer, and miR-130b played an important role in the regulation of pancreatic cancer malignant behavior including cell proliferation and invasion by directly targeting STAT3, indicating that miR-130b might be applied as a potential prognostic biomarker and inhibitor in pancreatic cancer [22]. Previously, Liu et al. reported that the expression level of miR-130b was upregulated in HCC tissues. Furthermore, circulating miR-130b in serum was a biomarker with clinical value for HCC screening [19]. However, whether miR-130b can serve as a prognostic biomarker of HCC has not been investigated. Therefore, we investigated the feasibility of miR-130b as a novel prognostic biomarker for HCC.

In the present study, our results showed that miR-130b expression was significantly higher in HCC tissues compared with normal adjacent liver tissues. The relationships of the miR-130b with various clinical features of HCC were analyzed. The results revealed that a high level of miR-130b expression was correlated with serum AFP level, HBsAg status, tumor size, high histologic grade, and high TNM stage, suggesting that miR-130b might be involved in the carcinogenesis and metastasis of HCC. Furthermore, the 5-year OS of high miR-130b expression group was significantly shorter than that of low miR-130b expression group. Moreover, the 5-year DFS of high miR-130b expression group was also significantly shorter than that of low miR-130b expression group. In a multivariate Cox model, we found that miR-130b expression was an independent poor prognostic factor for both 5-year OS and 5-year DFS, indicating that high miR-130b level might be a promising non-invasive biomarker for prognosis of patients with HCC.

## Conclusion

In conclusion, we demonstrated that miR-130b was significantly upregulated in HCC and correlated with poorer patients’ prognosis and it might be useful as a prognostic biomarker for HCC.
